# *In Vitro* Antidiabetic Effects of Isolated Triterpene Glycoside Fraction from* Gymnema sylvestre*

**DOI:** 10.1155/2018/7154702

**Published:** 2018-08-08

**Authors:** Rashmi S. Shenoy, K. V. Harish Prashanth, H. K. Manonmani

**Affiliations:** ^1^Department of Food Protectants and Infestation Control, CSIR-Central Food Technological Research Institute, Mysore 570020, India; ^2^Academy of Scientific and Innovative Research, CSIR-Central Food Technological Research Institute, Mysore 570020, India; ^3^Department of Biochemistry, CSIR-Central Food Technological Research Institute, Mysore 570020, India

## Abstract

A triterpene glycoside (TG) fraction isolated and purified from ethanolic extract of* Gymnema sylvestre *(EEGS) was investigated for blood glucose control benefit using* in vitro* methods. The HPLC purified active fraction TG was characterized using FTIR, LC-MS, and NMR. The purified fraction (TG) exhibited effective inhibition of yeast *α*-glucosidase, sucrase, maltase, and pancreatic *α*-amylase with IC_50_ values 3.16 ± 0.05 *μ*g/mL, 74.07 ± 0.51, 5.69 ± 0.02, and 1.17 ± 0.24 *μ*g/mL, respectively, compared to control. TG was characterized to be a mixture of triterpene glycosides: gymnemic acids I, IV, and VII and gymnemagenin.* In vitr*o studies were performed using mouse pancreatic *β*-cell lines (MIN6). TG did not exhibit any toxic effects on *β*-cell viability and showed protection against H_2_O_2_ induced ROS generation. There was up to 1.34-fold increase in glucose stimulated insulin secretion (p<0.05) in a dose-dependent manner relative to standard antidiabetic drug glibenclamide. Also, there was further one-fold enhancement in the expression of GLUT2 compared to commercial standard DAG (deacylgymnemic acid). Thus, the present study highlights the effective isolation and therapeutic potential of TG, making it a functional food ingredient and a safe nutraceutical candidate for management of diabetes.

## 1. Introduction

Type II diabetes is one of the most widespread metabolic disorders in the world, characterized by hyperglycemia and hyperlipidemia. About 190 million people are affected by diabetes, and the number is projected to rise to 642 million by 2040 [1]. It is known that the onset of diabetes is related to many classic risk factors including oxidative stress [2]. The complication associated with diabetes will be enhanced due to the involvement of reactive oxygen species (ROS) [3]. The ROS contributes to oxidative stress and in turn damages pancreatic islets and reduces insulin secretion. Growing evidences show that *β*-cells are central to the development of type 2 diabetes [4]. Therefore, the functional defects in *β*-cells are often accompanied by a reduction in expression of glucose transporter-2 (GLUT2) levels, further characterized by increased apoptosis of *β*-cells [5, 6]. Therefore, therapies for improving the *β*-cell function have become the potential new strategy to control hyperglycemia [7]. Studies demonstrate that supplementation with natural antioxidant products has shown improvement of hyperglycemic status by reducing the oxidative stress [8]. Also, glucose generation and absorption in the intestine play a vital role in hyperglycemia management [9]. Inhibition of *α*-glucosidase and *α*-amylase which digest dietary starch into glucose was studied as a method for controlling blood sugar levels [10]. Although several drugs for type II diabetes are* in vogue*, they have shown side effects such as liver toxicity and gastrointestinal adversity [11]. Thus the quest for safe, economical, and pharmacologically active molecules with multifunctional attributes towards diabetes management is necessary. This can be achieved by screening natural sources such as medicinal plants. Moreover, there is considerable interest in the application of traditional plants due to their natural origin, easily cultivable with minimum or no side effects.

*Gymnema sylvestre *(GS), a woody vine-like climbing plant, is well known in Indian traditional medicine “Ayurveda”. It grows in the tropical forest of central and southern India [12]. The leaves are used mostly as antiviral, diuretic, antiallergic, hypoglycemic, hypolipidemic, antibiotic, antianalgesic, and antirheumatic agents [13]. The leaves of GS contain triterpenoidal saponins belonging to oleanane and dammarane classes [13]. Twenty different saponins and glycosides have been reported in* Gymnema sylvestre *[14]. Several studies suggest that gymnemic acids may act as antidiabetic by promoting regeneration of islet cells and increase insulin secretion [15]. Also, gymnemic acids have been reported to inhibit glucose absorption from the intestine and utilize glucose by enhancing the activities of enzymes in insulin-dependent pathways [15].

The current focus of our study was to isolate and characterize bioactive agents (s) from* Gymnema sylvestre* and evaluate* in vitro* antidiabetic effects to initiate a search for nutraceutical pharmacophores towards inhibition of pancreatic *α*-amylase and *α*-glucosidase with improvement in beta cell function.

## 2. Materials and Methods

### 2.1. Chemicals

*α*-Amylase from porcine pancreas, *α*-glucosidase from* Saccharomyces cerevisiae*, para-nitrophenyl-glucopyranoside (*p*NPG), soluble starch, and 3-4,5-(dimethylthiazol-2-yl)-2,5-diphenyl tetrazolium bromide (MTT) were procured from SRL chemicals (Bangalore, India). Rat intestinal acetone powder (source of *α*-glucosidase), acarbose, Anti-GLUT2 antibody, anti-insulin antibody, 2′7′-dichlorodihydrofluorescein diacetate (DCFH-DA), acridine orange (AO), and ethidium bromide (EtBr) were from Sigma Chemical Co. (St. Louis, MO, USA). Glucose oxidase kit was purchased from AGAPE diagnostics (India). ELISA kits were purchased from Cytoglow Co. (India). Analytical and HPLC grade solvents were from E. Merck (India). The dried powder of* Gymnema sylvestre* was obtained from Nikhila Karnataka Central Ayurvedic Pharmacy (Mysore, India). Mouse pancreatic *β*-cell line (MIN6) was purchased from NCCS (Pune, India). Dulbecco's minimal essential medium (DMEM), Fetal Bovine Serum (FBS), and antimycotic solution were purchased from Himedia chemicals (India). All other chemicals used in the study were of analytical grade and purchased from Himedia chemicals (India).

### 2.2. Preparation of* Gymnema sylvestre* (GS) Extracts

GS powder was extracted using different solvent systems like methanol, ethanol, acetone, ethyl acetate, chloroform, and water, respectively. The GS powder along with respective solvents were left for 3 h refluxing, and the resulting infusions were filtered. The filtrates were concentrated; solvents were evaporated using rotary evaporator (Cole-Parmer SB 1100, Shanghai, China) at 45°C under reduced pressure and then lyophilized. The greenish-black powder thus obtained was stored at 4°C until used for further assays.

### 2.3. *α*-Glucosidase Inhibitory Assay

Extracts of GS were screened for *α*-glucosidase inhibitory activity according to the method described in [16]. The source of *α*-glucosidase enzyme was from* Saccharomyces cerevisiae*. The substrate solution* p*NPG (2 mg/mL) was prepared in 50 mM phosphate buffer, pH 6.8. 10 *μ*L of *α*-glucosidase (1.12 U/mL) was preincubated with 10 *μ*L of known concentrations of GS (extracted from different solvents) for 10 min.* p*NPG (10 *μ*L) was added to start the reaction followed by incubation at 37°C for 20 min. The reaction was stopped by adding 2 mL of 0.1 M Na_2_CO_3_. The *α*-glucosidase activity was determined by measuring the yellow-colored para-nitrophenol released from* p*NPG at 405 nm. The results were expressed as percentage of the blank control.

### 2.4. Preparative HPLC of Ethanolic Extract of* Gymnema sylvestre* (EEGS)

200 g of dried plant material was extracted using 1 L absolute ethanol under refluxing conditions for 3h. The extracted volume was concentrated using rotary evaporator (Cole-Parmer SB 1100, Shanghai, China) at 45°C at high pressure and brought down to 500 mL. This was further concentrated to ~5 g wet weight and a stock of 1 mg/mL was prepared using HPLC grade methanol solution. The stock solution was filtered using nylon 6 membrane consisting of six multiple filter papers with a pore size of 0.45 *μ*m, degassed by ultrasonic treatment before use. Preparative HPLC (gradient mode) was carried out using ODS C-18 silica column (25 cm X 21.2 mm, 12 *μ*m). The mobile phase was prepared using two solvent systems: solvent A: water (HPLC GRADE) containing 1 % acetic acid; solvent B: acetonitrile containing 1 % acetic acid. The mobile phase was filtered using nylon 6 multiple filter membranes having a pore size of 0.45 *μ*m. The conditions for HPLC [0 min solvent B (5 %), 5 min (8 %), 7 min (12 %), 12 min (18 %), 17 min (22%), 24 min (35 %), 26 min (100 %), 30 min (100 %), 32 min (5 %), 35 min stop] were set; constant flow rate was maintained at 5 mL/min. The detector was set at 254 nm, and fractions were collected. The fractions were made solvent free using a rotary evaporator, freeze-dried and then resuspended in known amount of phosphate buffer (50 mM, pH 6.8), and were analyzed for percentage *α*-glucosidase inhibitory activity.

### 2.5. Thin Layer Chromatography (TLC) and High Performance TLC (HPTLC)

The purity of the HPLC purified active fraction was tested by thin layer chromatography. Silica gel-G was taken as stationary medium and coated over glass slides. The mobile phase used was toluene:ethylacetate:acetic acid (5:7:1, v/v) [17]. Deacylgymnemic acid (DAG) was used as a standard. The purity of the active fraction was checked by HPTLC. HPTLC system equipped with a Linomat IV sample applicator, a twin-chamber tank, a model III TLC scanner, and CATS 4.0 integration software was employed. Aluminum-backed layers (10 × 10 cm) of silica gel GF254 were used as absorbent. The mobile phase used was chloroform and methanol (8:2, v/v). The spotted plates were developed up to 80 mm under chamber saturation conditions. After air-drying the solvent, the plates were scanned at 205 nm [18].

### 2.6. Characterization by FTIR, LC-MS, and NMR

The isolated active fraction was characterized by spectroscopic methods. FTIR (Bruker, USA) spectra were recorded in the range of 4000-400 cm^−1^ by blending samples with KBr. LC-MS (Shimadzu, Japan) was used to detect the molecular weights of the bioactive agents (s) detected in the active fraction. The separation was carried out using C18 column (150 x 4.6 mm ID, 5*μ*), column temperature maintained at 40°C. Mobile phase consisted of methanol and water both containing methanol and water (80:20) at a flow rate of 1 mL/min over 25 minutes. The electrospray ionisation was carried out in both positive and negative mode. ^1^H and ^13^C NMR spectra were recorded in DMSO-d_6_ on a Bruker Avance, USA, 400 MHz spectrometer (Bruker, Karlsruhe, Germany). Assignments of ^13^C signals were based on respective chemical shift values.

### 2.7. *α*-Amylase Inhibitory Assay

This assay was carried out using a modified procedure described in [19]. Briefly, a total of 100 *μ*L of different concentration of TG (250 ng–500 *μ*g /mL) was placed in a tube and 100 *μ*L of 50 mM sodium phosphate buffer (pH 6.8) containing *α*-amylase solution (1.5 mg/mL) was added. This solution was preincubated at 25°C for 20 min, after which 100 *μ*L of 1% soluble starch solution in 50 mM sodium phosphate buffer (pH 6.8) was added and incubated at 25°C for 10 min. The reaction was stopped by adding 500 *μ*L of dinitrosalicylic acid (DNS) reagent followed by incubation in boiling water for 5 min and then cooled to room temperature. The resulting mixture was diluted with 5 mL distilled water and the absorbance was measured at 540 nm. A control was prepared using the same procedure replacing the extract with distilled water. The *α*-amylase inhibitory activity was calculated as percentage inhibition. Concentrations of extracts resulting in 50% inhibition of enzyme activity (IC_50_) were determined graphically.

### 2.8. Rat Intestinal *α*-Glucosidase Inhibitory Activity (Sucrase and Maltase Inhibitory Activities)

The assay was based on the modified method described [20]. 100 mg of rat intestinal acetone powder was dissolved in 3 mL of NaCl (0.9 %) solution and centrifuged at 12,000 g for 30 min and used for the assay. The enzyme solution (30 *μ*L) was incubated with 40 *μ*L of sucrose (400 mM) for sucrase assay. Various concentrations of the extract were taken and made up to final volume of 100 *μ*l with 0.1 M phosphate buffer, pH 6.8. The reaction mixture was incubated at 37°C for 30 min. The reaction was then stopped by suspending mixtures in boiling water for 10 min. The concentrations of glucose released from the reaction mixtures were determined by glucose oxidase method with absorbance at a wavelength of 505 nm. Concentrations of extracts resulting in 50% inhibition of enzyme activity (IC_50_) were determined graphically.

Similarly, maltase assay was also carried out using the same protocol except that sucrose was replaced with 10 *μ*L maltose (86 mM).

### 2.9. Cell Culture

MIN6 cells were maintained under 5% CO_2_, 37°C in DMEM media supplemented with 10 % FBS, 2 mM L-glutamine, 100 U/mL of penicillin, and 0.1 mg/mL streptomycin.

#### 2.9.1. Cell Viability Assay

3-4, 5-(Dimethylthiazol-2-yl)-2,5-diphenyl tetrazolium bromide (MTT) assay was based on the protocol described [21]. MIN6 cells were seeded at a density of 2 × 10^4^ cells/well in 96-well plates for 24 h. Then the cells were treated with various concentrations of TG and EEGS (20–650 *μ*g) and incubated for 24 h. After the required period of treatment, MTT solution [10 *μ*L; 5 mg/mL MTT in phosphate-buffered saline (PBS)] was added with continued incubation for 3 h. After that DMSO was added and again incubated for 30 min in the dark. The cell viability was then recorded at 570 nm.

#### 2.9.2. Intracellular ROS

The quantification of the cellular antioxidant activity was determined according to the method described in [22]. To measure ROS levels, 2′7′-dichlorodihydrofluorescein diacetate (DCFH-DA) was used. The cells were seeded in a 96-well microtiter plate at a density of 5 X10^4^ cells/well. After 24 h, the cells were treated with 50 and 100*μ*g concentration of TG and EEGS and were further incubated for 24 h at 5% CO_2_, 37°C. Then 25 *μ*M DCFH-DA was added, and incubation was continued further for 1 h. The intracellular oxidation of DCFH-DA in the wells was measured using fluorescence microplate reader at excitation (488 nm) and emission (525 nm).

#### 2.9.3. Fluorescence Microscopy of MIN6 Cells

MIN6 cells were seeded at a density of 1×10^5^ cells/mL on poly-L-Lysine coated cover slips in 6 well plates containing DMEM for 24 h. H_2_O_2_ treated cells were used as control to show oxidative stress. Subsequently, the cells were treated with 50 and 100 *μ*g concentrations of TG devoid of H_2_O_2_ for 24 h. Cells were stained by double dye method using acridine orange (AO) and ethidium bromide (1:1). The images were then captured using a fluorescence microscope (Nikon, Tokyo, Japan) [23].

#### 2.9.4. Glucose Stimulated Insulin Secretion Assay

DMEM (Serum-free) without glucose, supplemented with 2 mM L-glutamine and 25 mM HEPES, pH 7.4, was used in this study. Glucose solution was prepared at different concentrations (1, 3, 5, 8, and 25 mM) and incubated at 37°C before use. MIN6 cells were seeded at a density of 1X10^6^ in 96-well plates for 24 h. Subsequently, the cells were treated with 50 and 100 *μ*g concentrations of TG. Prior to the insulin secretion assay, the cells were starved in Krebs-Ringer solution containing 0.1% bovine serum albumin (BSA) with 1 mM glucose for 1h, and the wells were washed twice with the same buffer. The cells were later incubated with Krebs-Ringer solution containing 3, 8, 15, and 25 mM glucose, 10 mM glibenclamide and 8 mM glucose, 30 mM KCl and 8 mM glucose for 1 h). Insulin secreted into the medium was measured using an ELISA kit [23].

#### 2.9.5. Measurement of GLUT 2 Levels

The surface translocation of GLUT2 was measured according to the protocols described [24]. The cells were treated with TG at 50 and 100 *μ*g/mL for 24 h. After treatment, the target GLUT 2 level in the cell lysate was measured using ELISA kit. The primary antibody specific to the target protein GLUT2 was added and allowed to bind to their target epitopes. The secondary antibody tagged with HRP which was specific to the primary antibody was added, and the detection of GLUT2 was done using TMB-H_2_O_2_ substrate at 450 nm.

### 2.10. Statistical Analysis

Results were represented as mean ± SD. Two-way ANOVA was employed to carry out statistical analysis using GraphPad prism, version 5.0. The experiments were conducted in triplicate. A confidence limit of p<0.05 was considered statistically significant.

## 3. Results

### 3.1. Extraction and Isolation of Bioactive Agents (s) from* Gymnema sylvestre*

**Figure 1:**
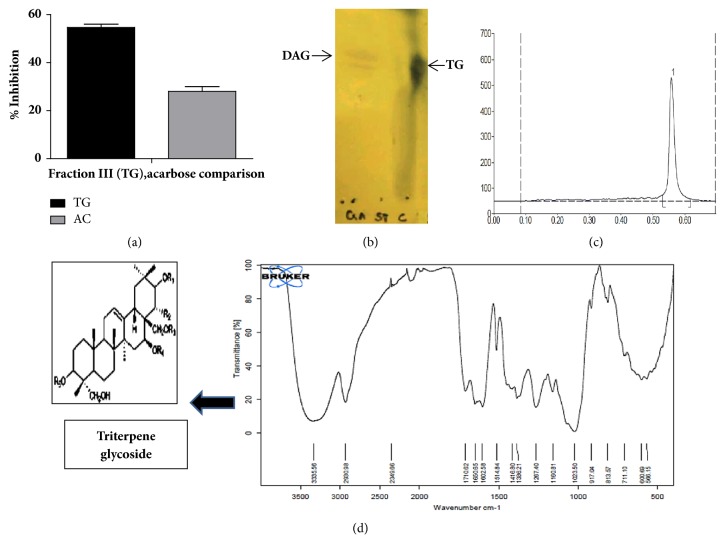
% *α*-glucosidase inhibition and identification of the active fraction TG. (a) shows the % *α*-glucosidase inhibition of the active fraction III (TG) relative to the positive control acarbose (AC). Values are expressed as mean ± SD. (b) represents the TLC pattern of the active fraction triterpene glycoside (TG labelled as C) and the available commercial standard deacylgymnemic acid (DAC labelled as GA); (c) represents the HPTLC pattern of triterpene glycoside (TG). The active fraction was checked for purity using TLC and HPTLC. TLC spot indicated the presence of gymnemic acid and HPTLC displayed rf value of 0.56. (d) depicts FTIR spectrum of TG. The characteristic FTIR peaks of TG with ester link 1710 cm-1 revealed presence of triterpene glycoside.

Dried leaf powder of GS (*Gymnema sylvestre*) was extracted using different solvents methanol, ethanol, acetone, ethyl acetate, chloroform, and water. All the solvent extracts were subjected to *α*-glucosidase inhibitory activity. Ethanolic extract showed maximum inhibition of 97.2 % (Table 1). This fraction was therefore chosen for further studies. The ethanolic extract of* Gymnema sylvestre *(EEGS) was subjected to chromatographic fractionation using preparative gradient HPLC system. The four peaks (fractions) obtained were collected, made solvent free, and resuspended in known amount of phosphate buffer (50mM, pH 6.8). *α*-Glucosidase inhibitory activity was assayed for all the collected fractions. Fraction III showed the highest inhibitory activity of 53.3% compared to standard acarbose (positive control) which exhibited 26% inhibition against *α*-glucosidase (Figure 1(a)). The other fractions showed lesser inhibitory activities ranging from 30 to 40% and hence fraction III was used for further studies. In the subsequent investigation, triterpene glycosides were identified in the active fraction III, after evaluation and characterization by TLC, HPTLC, FTIR, NMR, and LC-MS. This triterpene glycoside active fraction was termed as TG. The presence of gymnemic acid was indicated in TG by TLC (Figure 1(b)), when spotted along with the commercial standard deacylgymnemic acid (DAG). We also observed a single peak when TG was subjected to HPTLC with rf value 0.56 (Figure 1(c)).

**Figure 2:**
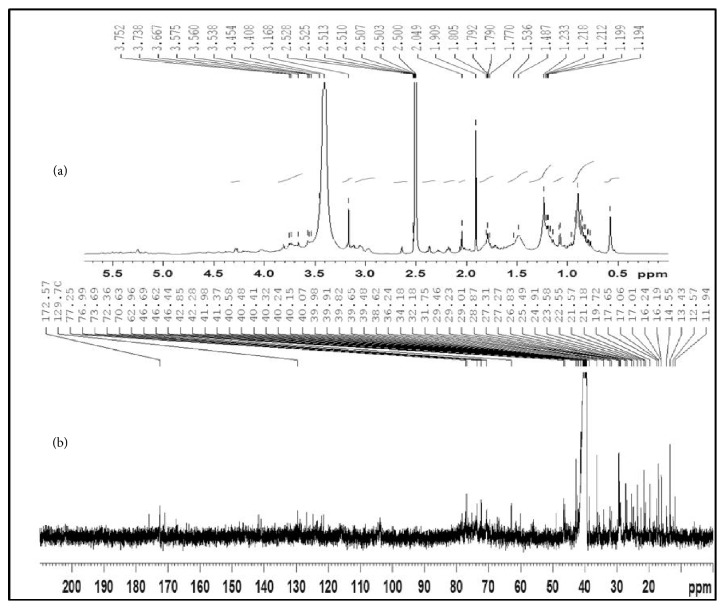
NMR spectrum of the active fraction TG. (a) 1H-NMR; (b) 13C-NMR.

FTIR spectrum showed the presence of bands at wavelengths 3335, 2930, and 1710 cm^−1^ corresponding to OH, CH, and C=O functional groups. The characteristic FTIR peaks of TG with ester link 1710 cm^−1^ (Figure 1(d)) revealed presence of typical gymnemic acid. To further elucidate the structure, TG was subjected to NMR assignments (Figure 2).  Table 2 gives the details of NMR chemical shifts assigned for TG.

**Figure 3:**
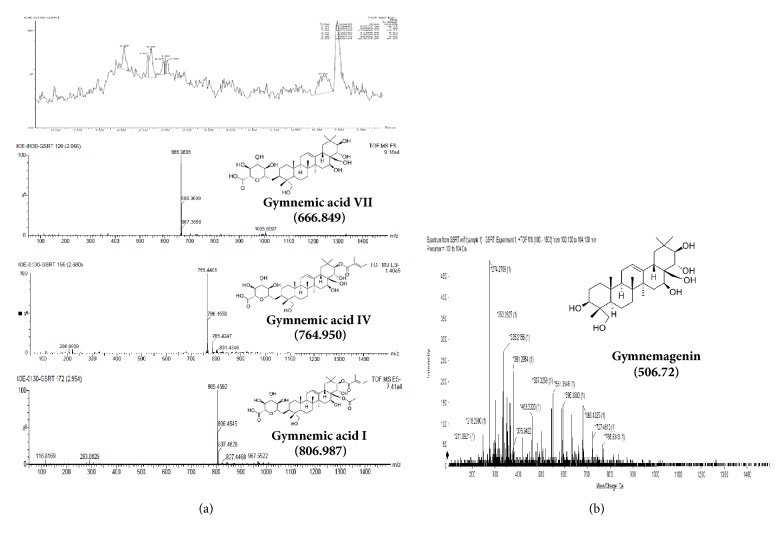
(a) LC chromatogram of TG, ESI -ve mode; (b) LC chromatogram of TG, ESI +ve mode.

^1^H NMR spectrum of TG was observed in DMSO–d_6_ solvent. The characteristic protons were found in their expected range (Figure 2). Further, the spectroscopic analyses employing chemical carbon (^13^C) shifts assignments (Table 2) were in good agreement with the MS negative mode data (Figure 3(a)). The spectrum suggested the presence of a tigloyl group at C-21 (Table 2) and acetyl group (172 ppm, –C=O & 23 ppm –CH_3_) at C-28 suggesting Gymnemic acid I. Saponins are classified as monodesmosides, with a single saccharide chain generally linked to C-3 depending on the number of sugars present. As usual, 77-76 ppm will appear for gymnemic acids due to glycosylation shifts. But we found 70 ppm for C-3 triterpenoids in their free form (sapogenins) indicating the presence of gymnemagenin. The presence of this compound was again reconfirmed with MS data in positive mode (Figure 3(b)). The sugar moiety was found to be 3-O-*β*-D-glucuronic acid (175ppm, –C=O). Moreover, the pattern gave a peak at NMR sugar region (103 ppm), and they appeared to consist of glucuronic acid alone and no other types of sugar moieties were present. Comparison in relative assignments revealed the presence of a mixture of gymnemagenin and gymnemic acids I, IV and VII, and they differ only in presence/absence of acetyl and tigloyl groups, respectively. These results are in good agreement with literature data with ± 1-2 ppm observed shifts which might be due to complex interactions in the above mixture of saponins in TG. Based on results obtained from ^1^H, ^13^C, and FTIR analyses, the structure of TG was proposed as shown in Figure 1(d).

In ESI -ve mode, the molecular weights were observed to be 765, 665, and 805, respectively. This indicated that TG contained gymnemic acids I (805), IV (665), and VII (765) all of which have similar triterpene backbone but differ in the positioning of functional groups. Under ESI +ve mode, peaks corresponding to triterpene backbone were observed. This indicated the presence of gymnemagenin, a triterpenoid aglycone of gymnemic acid with a molecular weight of 507 (m/z) (Figures 3(a) and 3(b)). These results correlate very well with the NMR patterns obtained.

### 3.2. Enzyme Inhibitory Activities

When subjected to different concentrations of TG, the inhibition of *α*-glucosidase was observed to be concentration dependent. This activity was higher compared to acarbose at their respective similar concentrations (Table 3). TG showed *α*-glucosidase inhibitory activity with an IC_50_ value of 3.16 ± 0.05 *μ*g/mL. Similarly *α*-amylase, sucrase, and maltase enzyme activities were inhibited by TG (Table 3) with IC_50_ values of 1.17 ± 0.24 *μ*g/mL, 74.07 ± 5.11 *μ*g/mL, and 5.69 ± 0.02 *μ*g/mL, respectively.

### 3.3. In Vitro Activity of TG Using MIN6 Cell (Pancreatic *β*-Cell) Lines

**Figure 4:**
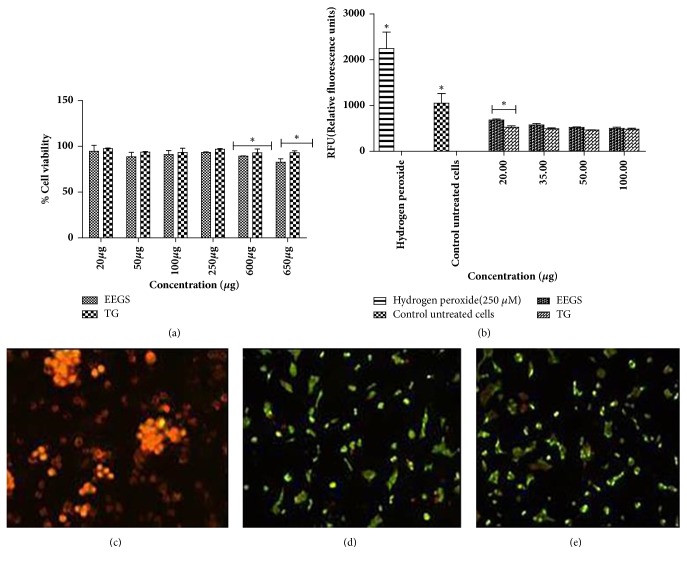
Effects of TG on *β*-cells viability. (a) MTT was performed to study the cytotoxic effects of TG. MIN6 cells were treated with different concentrations of TG and the cell viability was studied. (b) To study the ROS effect of GS extracts on MIN6 cells, treatment was carried out at concentrations 20, 35, 50, and 100 *μ*g levels. H_2_O_2_ was used as positive control. Fluorescent microscopy images showing the effect of TG on MIN6 cells, where (c) shows positive control (hydrogen peroxide), (d) shows TG treated cells (50 *μ*g), and (e) shows TG treated cells (100 *μ*g). Green regions indicate uptake of acridine orange, showing live *β* cells. Orange-red color displays apoptosis/dead cells. All values were represented as mean± SD. Experiments were performed in triplicate. Analysis was performed using two-way ANOVA. ^*∗*^Representing significance at p<0.05.

To examine whether the ethanolic extract of Gymnema sylvestre (EEGS) and its active fraction (TG) induced any cytotoxic effects, MTT assay was performed. When extracts were used at concentrations ranging from 20 to 650 *μ*g/mL, the viability retained up to 90% (p<0.05), even at highest concentration studied (650 *μ*g) (Figure 4(a)). This demonstrated the nontoxic effects of TG.

To further demonstrate that TG did not induce any cytotoxic effects, MIN6 cells were treated with TG and stained using differential staining method using AO and EtBr. AO/EtBr double staining combines the properties of two dyes to determine the type of cell death. The micrographs clearly indicate noncytotoxic nature of TG (50 and 100 *μ*g). The cells appeared green, and there was no decrease in cell number. H_2_O_2_ (200 *μ*M) treated cells appeared orange-red, and they were agglomerated indicating cell death due to toxicity (Figures 4(c), 4(d), and 4(e)).

The influence of TG on the generation of reactive oxygen species (ROS) was determined at 20, 35, 50, and 100 *μ*g/mL concentrations. H_2_O_2_ (200 *μ*M) treatment was used as control. A normal control without any treatment was also used. A significant reduction (p<0.05) in the formation of ROS was observed compared to H_2_O_2_ treated control at all the concentrations studied (Figure 4(b)). But the increase in sample concentration did not show any incremental significance.

### 3.4. Insulin Secretion by *β*-Cells

**Figure 5:**
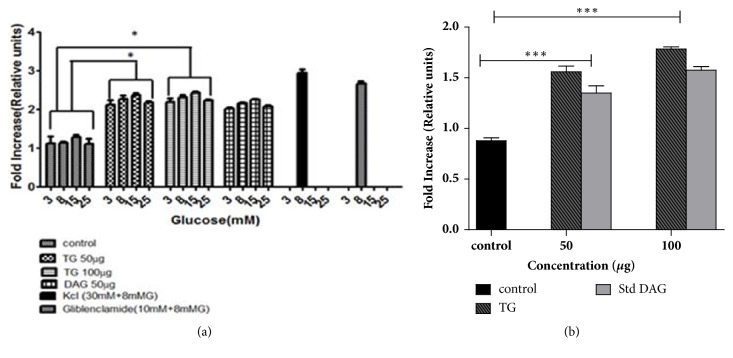
Effect of TG on* in vitro *antidiabetic activity. (a) MIN6 cells were treated with TG at 50 and 100*μ*g concentrations. Deacylgymnemic acid (DAG), an available commercial standard from GS, and glibenclamide, a commercial drug used for diabetes treatment, were also used for comparison. (b) represents effect of TG on enhancement of GLUT2 levels. Treatment with TG and standard DAG (from GS) was done at 50 and 100 *μ*g concentrations. All experiments were carried out in triplicate and results were represented as mean± SD. Analysis was performed using two-way ANOVA. ^*∗*^Representing significance level at p<0.05. ^*∗∗∗*^Representing significance level at p<0.001.

The *β*-cells displayed dose-dependent response in insulin secretion when stimulated with increasing concentrations of glucose (3-25 mM) in the presence of 50 and 100 *μ*g concentrations of TG. TG at 50 *μ*g level displayed 1.0-fold increase (p<0.05) in insulin secretion when cells were exposed to a lower concentration of glucose (3mM and 8mM) compared to the control. The glucose concentrations 5 and 25 mM were considered as normoglycemic and glucotoxic, respectively. But the cells incubated with normoglycemic glucose levels showed a little rise in insulin levels (1.34-fold, p<0.05). At glucotoxic conditions, the response was quite lower than that of normoglycemic glucose levels yet significant compared to the control, respectively, as illustrated in Figure 5(a) (1.16-fold, p<0.05). When cells were treated with TG at 100 *μ*g at low concentrations of glucose (3mM), there was 1.12-fold increase in insulin level (p<0.05) compared to control. At normoglycemic and glucotoxic glucose levels, the response of the cells was similar to TG at 50 *μ*g level in insulin secretion as shown in the Figure 5(a). The results obtained were comparable with the positive controls (KCl and glibenclamide) used in this study.

### 3.5. Effect of TG on GLUT2

We examined the effect of TG on GLUT2 by quantitative ELISA using anti-GLUT2 antibody tagged to HRP (Figure 5(b)). TG treated cells demonstrated better increase in GLUT-2 levels at 50 and 100 *μ*g concentrations compared to commercial standard DAG (deacylgymnemic acid). Preincubation of cells at 50 *μ*g of TG showed a slight increase in GLUT2 levels (0.5 fold), yet it was significant compared to control (p<0.01). When cells were treated with TG at 100 *μ*g, a single fold increase in GLUT2 levels was observed when compared to control (p<0.001).

## 4. Discussion

Pancreatic *α*-amylase breaks down complex carbohydrates into oligosaccharides and disaccharides. Further, the intestinal *α*-glucosidases digest diet-derived oligosaccharides and disaccharides into monosaccharides, mainly glucose. In previous reports, the ethanolic extract of* Gymnema montanum* leaves demonstrated appreciable *α*-glucosidase and *α*-amylase inhibitory activity comparable to that of acarbose, a commercially available drug that inhibits *α*-glucosidase to decrease postprandial hyperglycemia [25]. In the present study, we have observed that isolated active fraction TG inhibited the activity of pancreatic *α*-amylase, intestinal *α*-glucosidases (sucrase and maltase), and yeast *α*-glucosidase in a concentration-dependent manner as shown in the Table 3. The inhibition effect was significant (p<0.05) against intestinal *α*-glucosidase (sucrase) with an IC_50_ value of 74.07 ± 0.51 *μ*g/mL relative to acarbose, which showed IC_50_ value of 44.30 ± 0.73 *μ*g/mL. We found promising inhibition with IC_50_ value for TG (3.16 ± 0.05 *μ*g/mL) against yeast *α*-glucosidase compared to acarbose. Similar effects were observed* in vivo, *where inhibition of *α*-glucosidase and other intestinal enzymes such as maltase and sucrase occurs in polysaccharides and oligosaccharides digestion [26]. *α*-Amylase and *α*-glucosidase showed different inhibition patterns probably due to structural differences related to the origins of the enzymes [27]. Gymnemic acid (GA) isolated from GS not only inhibits glucose absorption in the small intestine [27], but also suppresses hyperglycemia and hyperinsulinemia in an oral glucose tolerance test [28]. The efficiency of GA in inhibiting glucose absorption in the small intestine was found to be increased by a combined effect with acarbose and voglibose [28]. Thus we have attempted to study antihyperglycemic effect induced by TG* in vitro,* since it seems to be little more feasible than that* in vivo*. The inhibition of these enzymes could be probably because of the synergistic action of triterpene glycosides present in the active fraction TG as substantiated by* in vitro* antidiabetic studies. Bekzod Khakimov et al. (2016) [29] have shown in their study the mass spectral fragmentation patterns of 49 saponin peaks, detected from plant originating from eight different triterpenoid aglycones with different MW. Further, they have shown that continuously measured proton NMR data during chromatography separation along with mass spectrometry data revealed significant differences, including contents of saponins, types of aglycones, and numbers of sugar moieties attached to the aglycone. The review [30] has also showed the results about the isolation and chemistry of the triterpenoids and their physicochemical characterization will be better studied using MS, FTIR, and NMR spectral data.

Previous study showed that the aqueous extract of GS decreased blood glucose levels in rats and humans, without improving insulin sensitivity [31]. An insulin secretagogue function was ascribed to GS following observations that its administration led to elevations in serum insulin levels in individuals with type 2 diabetes [32]. However, to identify the mode of action for glucose-lowering agents, we have carried out experiments* in vitro* for potential effects of GS extracts to stimulate insulin secretion by direct action at *β*-cells. Pretreatment with (50−100 *μ*g) of TG for 2 h triggered a significant increase of glucose-induced insulin secretion in a dose-dependent manner. This may be mainly attributed to the effect of TG on the insulin secretion enhancement capacity of MIN6 cells. The ability of TG to further stimulate insulin secretion at 15 mM glucose demonstrated that TG acts by enhancing glucose metabolism within the *β*-cells. TG was also an efficient amplifier of insulin secretion, thus assisting in sustained amplification of insulin secretion at 15 and 25 mM glucose concentrations, respectively. The ability of TG to stimulate insulin secretion at substimulatory glucose concentrations was comparable to glibenclamide, a standard drug for stimulating insulin secretion used in our study. GS has been widely utilized in Indian medicine (Ayurveda) for the treatment of diabetes and other disorders [33]. GS extracts contain various biologically active compounds that exert antidiabetic effects [34]. GS leaves contain numerous saponin components, some of which may solubilize mammalian cell membrane proteins in much the same way as digitonin, which was used experimentally to permeabilize plasma membranes [35]. The major saponins present in the isolated active fraction TG showed the stimulatory effect on insulin release from MIN6 *β*-cells at 50 and 100*μ*g, accompanied by 90-95% cell viability as revealed by staining with 1:1 mixture of ethidium bromide (EtBr) and acridine orange (AO). In one of the studies, novel isolate of GS termed OSA (named after Om Santal Adivasi) showed insulinotropic activities on *β*-cell lines and isolated human islets* in vitro *had beneficial effects on glycemic control in patients with type 2 diabetes [36]. OSA at around 0.25 mg/mL stimulated insulin secretion* in vitro* without adverse effects on cell viability. Similarly, in our study, TG at 50 *μ*g/mL level stimulated insulin secretion* in vitro* without hostile effects on cell viability. Even at 650 *μ*g/mL concentration tested, 90 % of *β*-cells were viable. These investigations suggested that membrane damaging components in TG or their concentrations might be much lower. Differential staining with ethidium bromide and acridine orange was an established test of cell viability that paves the way for rapid visual readout of compromised plasma membrane integrity. In this study increased dye uptake of acridine orange to give green fluorescence indicated the nontoxic effects of TG on *β*-cells.

The pancreatic islet includes the most vulnerable cells for the attack after ROS generation in human as it has less antioxidant enzymes as compared to other organs [8]. The damage of pancreatic islet leads to decrease in insulin secretion, resulting in hyperglycemic conditions and regeneration of ROS.* In vitro,* GS alcoholic leaf extract showed antioxidant ability by inhibiting 1,1-diphenyl-2-picrylhydrazyl (DPPH) and scavenging superoxide and hydrogen peroxide [37]. In the present study, H_2_O_2_ was chosen as known model biological ROS. A significant reduction in the formation of ROS was observed compared to control at all the concentrations of TG studied.

Many medicinal plants are used in the traditional medicine to enhance the translocation of GLUT, and this could lead to a new approach for treating type 2 diabetes. GLUT2 is a facilitative glucose transporter located in the plasma membrane of pancreas, liver, intestinal, kidney cells, and the hypothalamus areas [38]. Due to its high capacity and low affinity, GLUT2 transports dietary sugars, glucose, fructose, and galactose in a broad range of physiological concentrations. GLUT2 has the highest capacity and the lowest affinity for glucose, which allows glucose uptake in the beta cells only when the glucose level is high and insulin secretion is necessary [38]. According to literature, ethanolic extract of* Catharanthus roseus* enhanced GLUT2 levels in streptozotocin-induced diabetic Wistar rats. In our studies, at 50 *μ*g concentration of TG, there was 0.5-fold increase of GLUT2 compared to control. At 100*μ*g concentration, the increase was 1-fold (p<0.05). In a similar previous study conducted, methanolic leaf extract of* Gymnema sylvestre* (MLGS) demonstrated a significant and dose-dependent increase in glucose uptake in all the concentrations of MLGS studied [39]. TG at 50 and 100 *μ*g elevated GLUT2 levels comparable to standard DAG (deacylgymnemic acid). Renewal of glucose transport induction would, therefore, enhance the uptake of glucose and thus help to combat hyperglycemic conditions.

## 5. Conclusion

In conclusion, the present study exhibited an evidence that isolated active fraction, triterpene glycoside, TG from* Gymnema sylvestre*, could inhibit the activity of pancreatic *α*-amylase, *α*-glucosidase, sucrase, and maltase. Further, it was shown to enhance the GLUT2 protein levels and ameliorate impaired insulin secretion of MIN6 cells. However, the underlying molecular mechanism remains to be understood. The isolated and characterized active fraction, triterpene glycoside (TG), gives insight into dietary antidiabetic benefits by reducing antinutritional factors of saponins in* Gymnema sylvestre*.

## Figures and Tables

**Table 1:** % *α*-glucosidase inhibition for different solvent extracts of GS.

Solvents	% *α*-glucosidase Inhibition

Methanol	52.2 ± 0.50

**Ethanol**	**97.24 ± 0.24**

Acetone	80.34 ± 0.63

Ethyl acetate	7.1 ± 0.05

Chloroform	75.24 ± 0.40

Water	-

Known amount of GS powder was extracted using different solvent systems.The experiments were done in triplicate and average values were determined. Values are expressed as mean± SD.

**Table 2:** ^13^C NMR spectral data for gymnemagenin and respective gymnemic acids (*δ* in DMSO-d_6_).

**Position**	**Gymnemagenin (** **δ** **)**	**Gymnemic acid I (** **δ** **)**	**Gymnemic acid IV (** **δ** **)**	**Gymnemic acid VII (** **δ** **)**
C-1	38.6	++	++	38.6

C-2	27.2	26.8	26.8	27.2

C-3	70.6	77.3	76.5	76.5

C-4	42.3	42.9	42.3	42.3

C-5	49.1	-* *-	-* *-	49.1

C-6	17.7	17.6	17.6	17.7

C-7	32.2	-* *-	-* *-	32.2

C-8	40.2	++	++	40.2

C-9	46.7	46.6	46.6	46.7

C-10	36.2	++	++	36.2

C-11	24.9	24.5	23.6	24.9

C-12	121.5	124.8	122.2	121.5

C-13	141.8	-* *-	-* *-	141.8

C-14	41.9	-* *-	-* *-	41.9

C-15	34.2	35.6	35.6	34.2

C-16	67.3	-* *-	-* *-	67.3

C-17	46.0	46.0	46.4	46.0

C-18	42.0	-* *-	-* *-	42.0

C-19	46.4	-* *-	-* *-	46.4

C-20	34.2	36.0	36.0	34.2

C-21	77.0	78.4	78.4	77.0

C-22	73.7	72.5	72.5	73.7

C-23	70.6	62.3	62.3	70.6

C-24	13.4	14.6	14.4	13.4

C-25	16.2	16.3	16.3	16.2

C-26	17.0	17.1	17.1	17.0

C-27	27.2	27.3	27.3	27.2

C-28	60.0	63.0	60.0	60.0

C-29	31.8	29.5	29.2	31.8

C-30	19.7	21.6	21.2	19.7

		Acetyl 1 - 172.6		

		Acetyl 2 – 22.5		

		Tigloyl 1' - 170.9		
		Tigloyl 2' - 129.7		
		Tigloyl 3' - 139.1		
		Tigloyl 4' - 14 .4		
		Tigloyl 5' -12.6		

		**3-0-** **β** **-D-glucopyranosiduronic acid**		
		C1 - 103.9		
		C2 - 74.0		
		C3 - 76.6		
		C4 - 72.4		
		C5 - 75.2		
		C6 - 175.9		

++: overlapped signals; -* *-: not assigned.

**Table 3:** IC_50_ values for porcine *α*-amylase, yeast *α*-glucosidase, and mammalian *α*-glucosidase inhibitory potential of *TG.*

Enzymes	IC_50_ values for TG (*µ*g/mL)	IC_50_ values for acarbose (*µ*g/mL)
*α*-glucosidase (yeast source)	3.16 ± 0.05	6.14 ± 0.05

rat Intestinal *α*-glucosidase (sucrase)	74.07 ± 0.51^*∗*^	44.30 ± 0.73^*∗*^

rat Intestinal *α*-glucosidase (maltase)	5.69 ± 0.02	0.059 ± 0.05

porcine *α*-amylase	1.17 ± 0.24	0.18 ± 0.02

At different concentrations of TG IC_50_ values for yeast *α*-glucosidase, sucrase, maltase, and porcine *α* amylase were studied. Values are expressed as mean± SD of three independent experiments made in duplicate.

^*∗*^Indicating the significant levels at p<0.05.

## Data Availability

All the data used to support the findings of this study are included in the manuscript.

## Abbreviations

GS: * Gymnema sylvestre*
EEGS: Ethanolic extract of* Gymnema sylvestre*
*p*NPG: Para-nitrophenyl-glucopyranoside
TLC: Thin layer chromatography
HPTLC: High performance thin layer chromatography
HPLC: High performance liquid chromatography
FTIR: Fourier transform infrared spectrum
LC-MS/MS: Liquid chromatography mass spectrometry
NMR: Nuclear magnetic resonance
TG: Active fraction III, mixture of triterpenoid glycosides
MIN6: Pancreatic *β*-cell lines from mouse
MTT: 3-4,5-(Dimethylthiazol-2-yl)-2,5-diphenyl tetrazolium bromide
ROS: Reactive oxygen species
DCFH-DA: 2′7′-dichlorodihydrofluorescein diacetate′′′′′′
GLUT2: Glucose transporter 2.

## Supplementary Materials

Supplementary Materials The graphical representation depicts the gist of entire manuscript. In this work, we have attempted to evaluate the antidiabetic potential of Gymnema sylvestre. The active fraction “TG” has been characterized and explored for its antidiabetic effects. The active fraction TG showed inhibition of the disaccharidase activity, owing to its glucose control benefits. TG also improved insulin secretion and glucose transporter protein levels in beta cells in vitro.
