# Supplementary material for: In Vitro Antidiabetic Effects of Isolated Triterpene Glycoside Fraction from Gymnema sylvestre
Source: Evid Based Complement Alternat Med. 2018 Aug 8;2018:7154702. doi: 10.1155/2018/7154702 (PMC6106959; doi:10.1155/2018/7154702)
Supplement: Supplementary Materials — The graphical representation depicts the gist of entire manuscript. In this work, we have attempted to evaluate the antidiabetic potential of Gymnema sylvestre. The active fraction “TG” has been characterized and explored for its antidiabetic effects. The active fraction TG showed inhibition of the disaccharidase activity, owing to its glucose control benefits. TG also improved insulin secretion and glucose transporter protein levels in beta cells in vitro. [file 7154702.f1.pdf]

Graphical abstract

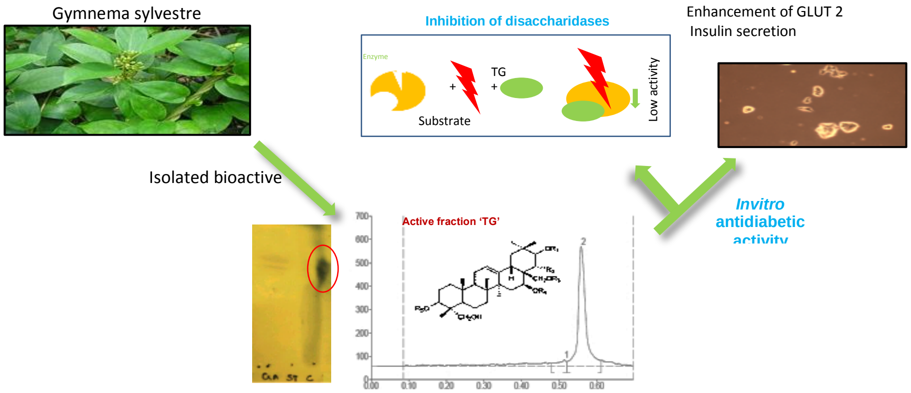

The above graphical representation depicts the gist of entire manuscript. In this work, we have attempted to evaluate the antidiabetic potential of *Gymnema sylvestre*. The active fraction 'TG' has been characterized and explored for its antidiabetic effects. The active fraction TG showed inhibition of the dissacharidase activity, owing to its glucose control benefits. TG also improved insulin secretion and glucose transporter protein levels in beta cells *in invitro.*

Highlights

- An active fraction from *Gymnema sylvestre* was isolated and characterized with reduced anti-nutritional factors.
- *in vitro* antidiabetic activity was performed to initiate a search for nutraceutical pharmacophores towards inhibition of pancreatic α-amylase & α-glucosidase .
- Study revealed increased cell viability, reduced oxidative stress, improvement in beta cell function.
- This study highlights the phytotherapeutic potential of active fraction, making it a alternate medicinal candidate for management of diabetes.
